# Oral Rehabilitation of a Case of Amelogenesis Imperfecta with Multiple Periapical Cysts

**DOI:** 10.5005/jp-journals-10005-1005

**Published:** 2008-12-26

**Authors:** Lini Mathew, Amitha M Hegde, Y Rajmohan Shetty

**Affiliations:** 1 Former Postgraduate Student, Department of Pedodontics and Preventive Children Dentistry, AB Shetty Memorial Institute of Dental Sciences, Derlakatte, Mangalore, Karnataka, India; 2 Professor and Head, Department of Pedodontics and Preventive Children Dentistry, AB Shetty Memorial Institute of Dental Sciences, Karnataka, India; 3 Professor, Department of Pedodontics and Preventive Children Dentistry, AB Shetty Memorial Institute of Dental Sciences, Karnataka, India

**Keywords:** Amelogenesis imperfecta, periapical cysts, surgical root canal treatment, occlusal rehabilitation.

## Abstract

Amelogenesis Imperfecta is a hereditary anomaly that affects the enamel of human teeth and is not associated with any systemic disorder of affected patients. The affected teeth are disturbed in coloration, thickness and resistance. The rehabilitation of amelogenesis imperfecta in a child must take into account the development of the child’s teeth, the health of the periodontal tissues and the maxillary and mandibular growth. This article reports the endodontic and occlusal rehabilitation of a 14-year-old girl affected with autosomal recessive hypocalcified type of amelogenesis imperfecta with multiple periapical cysts.

## INTRODUCTION

The term amelogenesis imperfecta has been defined to include a variety of genetically determined disorders that primarily affect the enamel of all or nearly all the teeth without causing detectable alteration elsewhere in the body.^1^ Thus amelogenesis imperfecta represents an inherited group of anomalies of enamel formation with an incidence of 1:718 to 1:14,000.^2^

At least 14 different subtypes of amelogenesis imperfecta exist with numerous patterns of inheritance and a variety of clinical manifestations. On the clinical and radiographic basis, three broad types exist, i.e. hypoplastic, where the enamel is reduced in quantity but is relatively well mineralized.

Hypocalcified, where the enamel is formed in normal amounts but is poorly mineralized. 

Hypomaturation, where the final stage of mineralization stage is abnormal.^2^

The clinical features distinguish the three types.^1^,^3^

- Hypoplastic-The enamel does not form in normal thickness.
- Hypocalcified-Enamel thickness on newly erupted teeth closely approaches that of normal teeth, but the enamel is soft, friable and can be easily removed from the dentin.
- Hypomaturation type-Develop enamel of normal thickness. The hypomaturation type differ from hypocalcification in that enamel is harder, with a mottled opaque white to yellow brown to a red brown color and tend to chip from the underlying dentin rather than wear away.

Restoration of these defects is important not only because of aesthetic and functional concerns, but also there may be a positive psychological impact for the patient.^4^ Treatment planning for patients with amelogenesis imperfecta is related to many factors: the age and socioeconomic status of the patient, the type and severity of the disorder, the intraoral situation at the time of treatment planned.^5^ If teeth affected with amelogenesis imperfecta are not detected and treated early; further deterioration of the existing condition will occur with damage to the periodontal tissues, thus further complicating the treatment plan and prognosis of the patient. In the present case, we tried to rehabilitate a child with amelogenesis imperfecta whose oral condition had deteriorated due to poor early management.

## CLINICAL REPORT

A female patient aged 14 years came to the Department of Pediatric Dentistry, A B Shetty Institute of Dental Sciences, Mangalore with a complaint of discolored teeth since its eruption and intermittent pain in the front region of the upper and lower jaw which aggravated on biting since the last three years. The primary dentition was also reported to be discolored by the patient. The medical history revealed that the patient was partially blind since birth which was diagnosed to be due to atrophic damage to the optic nerve. The patient also gave a history of anxiety bordering on hysteria for which she was undergoing counseling. She was not on any medication for either of her medical conditions. The family history revealed that her parents had a consanguineous marriage (1st cousins) and there was no history of any other members of the family having a similar discoloration of the teeth.

**Fig. 1:**
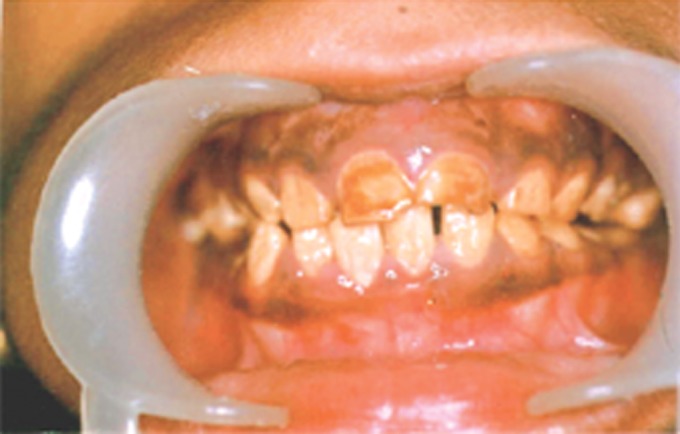
Dentition affected with autosomal recessive hypocalcified type of amelogenesis imperfecta

The intraoral examination revealed that the permanent dentition was present with inflammation and redness in the attached gingiva in relation to the upper left lateral incisor and a sinus opening in relation to lower right central incisor. The oral hygiene was poor with accumulation of plaque and calculus. The teeth were brownish yellow in color and tissue loss affected nearly all the permanent teeth. The enamel was so soft that much of it was soon lost after eruption leaving the crown composed only of dentin. The enamel had a cheesy consistency and could be scraped off the dentin with a sharp instrument (Fig. 1). Translucency of enamel could not be demarcated and generalized attrition with loss of proximal contacts and vertical dimension was seen. The occlusal review revealed a skeletal class II pattern with increased overjet and overbite and shift of the mandible towards the right by 4 millimeters.

**Fig. 2:**
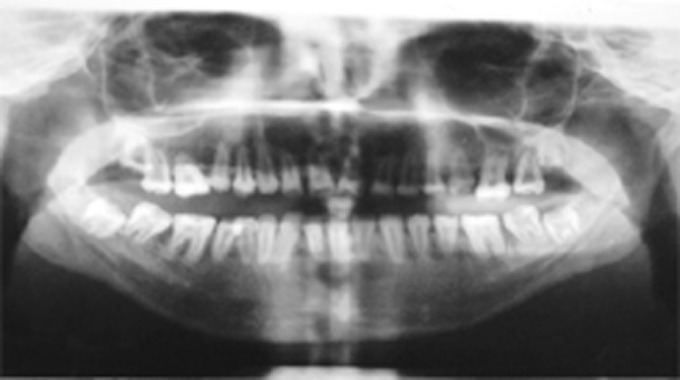
OPG of the dentition affected with hypocalcified type of amelogenesis imperfecta

**Figs 3A to E:**
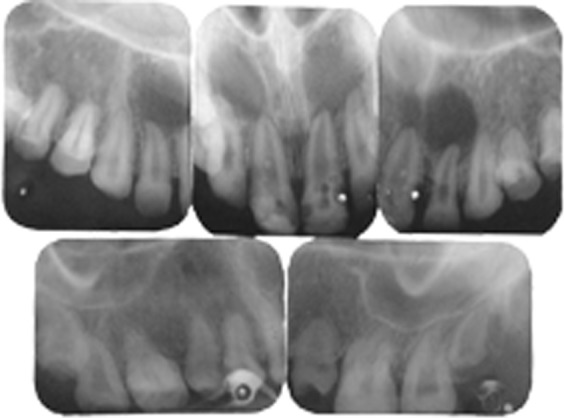
IOPAR of dentition in upper arch showing periapical radiolucency in relation to upper right and left central and lateral incisors

**Figs 4A to E:**
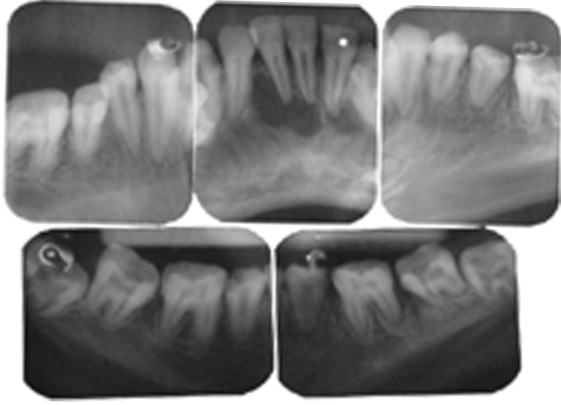
IOPARs of the dentition in the lower arch showing periapical radiolucency in relation to lower right and left central and lateral incisors

Radiographic examination revealed that the radiodensity of enamel was lesser than that of dentin and enamel could be appreciated only on the cervical areas on most of the teeth (Fig. 2). Well demarcated radiolucent lesions were observed in relation to the periapical regions of upper right and left central and lateral incisors and lower right and left central and lateral incisors (Figs 3 and 4A to E). Routine blood and urine examination and assessment of blood calcium, phosphate and alkaline phosphatase was done and was found to be within normal limits. On the basis of family history, clinical and radiographic picture and lab investigations she was diagnose to have an isolated case of autosomal recessive hypocalcified type of amelogenesis imperfecta^2^ with multiple periapical cysts. This patient had earlier been treated only with antibiotic therapy by two dentists without treating the pulp pathology or taking into consideration the pathology of the dental hard tissues, which further deteriorated the condition.

The treatment of children with amelogenesis imperfecta consists of three major phases

- Phase I—Temporary emergency treatment of primary or permanent teeth
- Phase II—Interim or transitory treatment of permanent teeth
- Phase III—Treatment of adulthood.^6^

The treatment plan must keep in mind the goals of the patients and parents in order to arrive at a reasonable plan.^7^ After discussing the various treatment options with the patient and parents and getting an informed consent from the parents, it was decide to treat the patient in two phases. Phase I involved the surgical root canal treatment of the affected teeth and phase II consisted of the occlusal rehabilitation with full coverage temporary-permanent restorations as the patient was still in the transitional stage of growth. Phase III with permanent occlusal rehabilitation will be done only after the maxillary and mandibular growth has ceased.

### Phase I (Endodontic Rehabilitation)

**Figs 5A to D:**
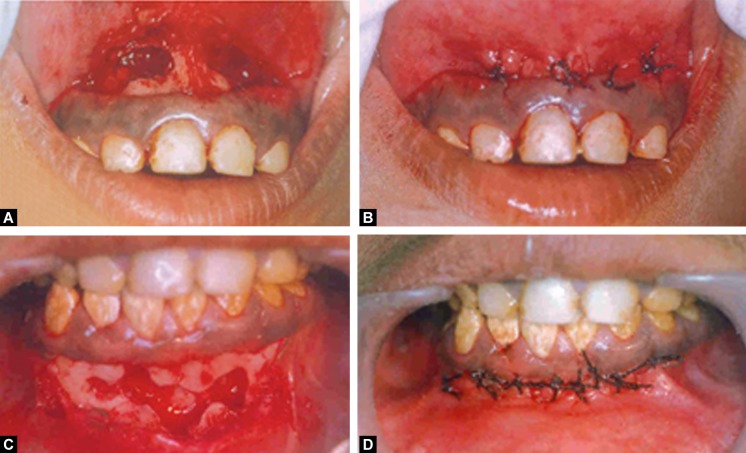
Surgical root canal treatment done in relation to the upper and lower anterior teeth

**Figs:6:**
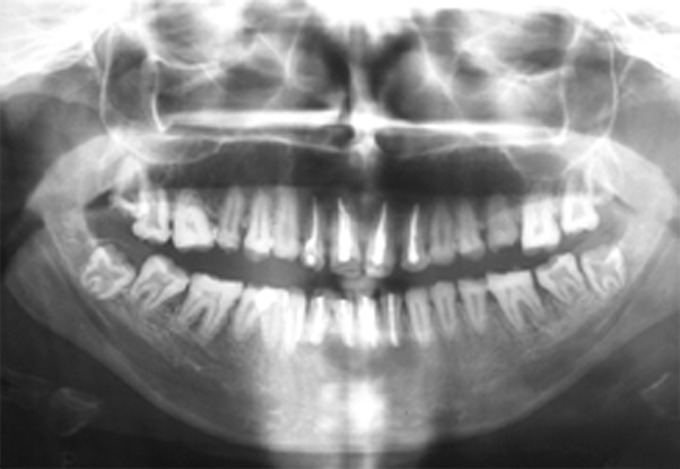
OPG after surgical root canal treatment

**Figs 7A and B:**
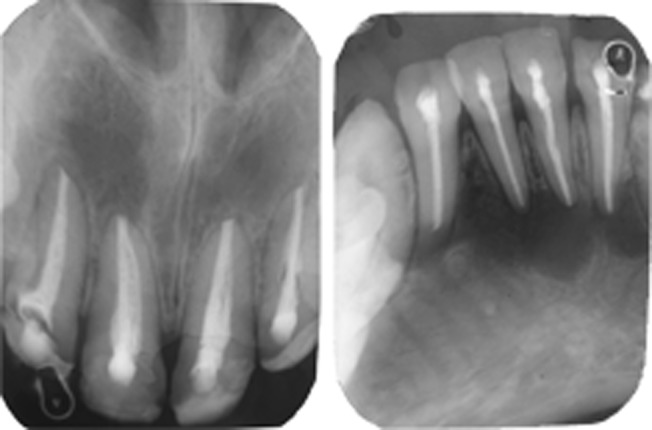
IOPARs of upper and lower anterior teeth after surgical root canal treatment

The behavior of the patient was cooperative; hence it was decided to do the surgical root canal treatment under local anesthesia. Periapical surgery was performed in relation to the affected teeth with an interval of two weeks between the treatment of the teeth in the upper and lower arches. The necrotic pulp was extirpated in relation to the affected teeth and a thin mix of Ca(OH)_2_ was given as an intracanal medicament fortnightly for three visits till the seepage of the canals reduced. After six weeks the flap was raised, the periapical lesion was enucleated, the root end of the affected teeth were resected and the canals were obturated with gutta percha.The gutta percha was cold burnished at the apical ends and the exposed dentinal margins were sealed with glass ionomer cement [(GC corporation, Tokyo, Japan), (Figs 5A to D to 7A and B)]. The postoperative period was found to be uneventful. The excised tissue was subjected to histopathological examination and it was confirmed to be periapical cysts.

### Phase II (Occlusal Rehabilitation)

**Figs 8 A to D:**
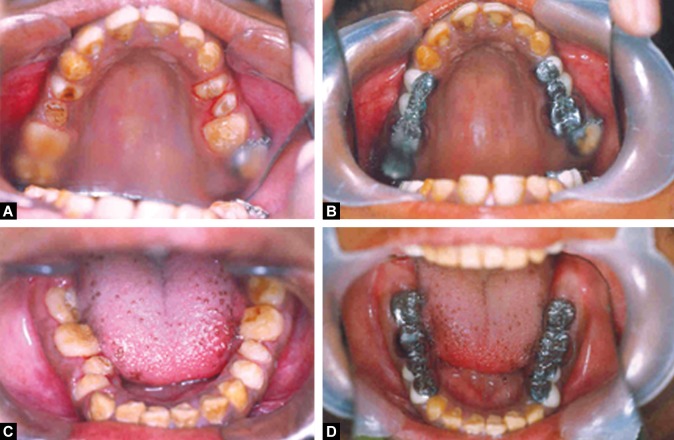
Crown preparation done on the upper and lower posterior teeth and cast crowns given

**Figs 9A to D:**
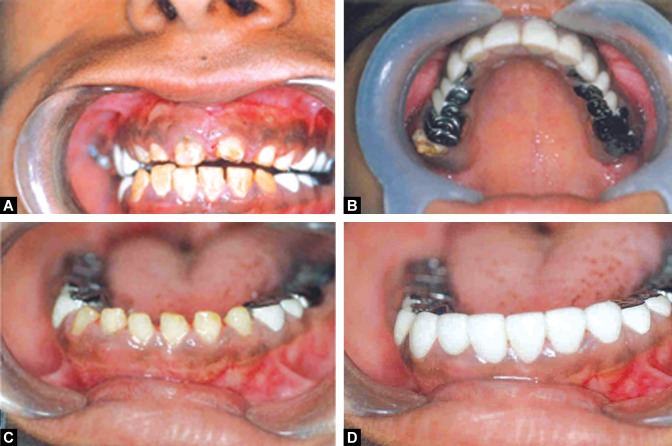
Crown preparation done on the upper and lower anterior teeth and full coverage acrylic crowns given

A month after the endodontic rehabilitation, the diagnostic casts of the upper and lower arches and interocclusal records were made .The crowns of the upper and lower posterior teeth were prepared. As maxillary right second molar was placed buccally on the dental arches and the patient was not willing for orthodontic treatment, it was decided not to include this tooth in the crown preparations. Provisional restorations with tooth colored self curing acrylic resin were given with an increase of the vertical dimension by 2 mm which was well within the freeway space. The patient was monitored over a two month period to check for any functional or articulatory problems following the change in vertical dimension of occlusion. Then the provisional restorations were replaced with cast metal crowns (Ni-Cr) on the molars and acrylic fused to metal full coverage crowns (Ni-Cr) at the earlier established vertical dimension of occlusion (Figs 8A to D) as temporary-permanent restorations. Preparation of the crowns of the upper and lower anterior teeth was done at the same visit and provisional restorations with tooth colored self curing acrylic resin was given. At the next visit the anterior teeth were restored with full coverage acrylic crowns establishing an anterior guidance that disoccluded the posterior teeth in all eccentric excursions, i.e. in protrusive, lateral and laterotrusive (Figs 9A to D). All the crowns were cemented with glass ionomer luting cement (GC, corporation, Tokyo, Japan).

**Figs 10 A and B:**
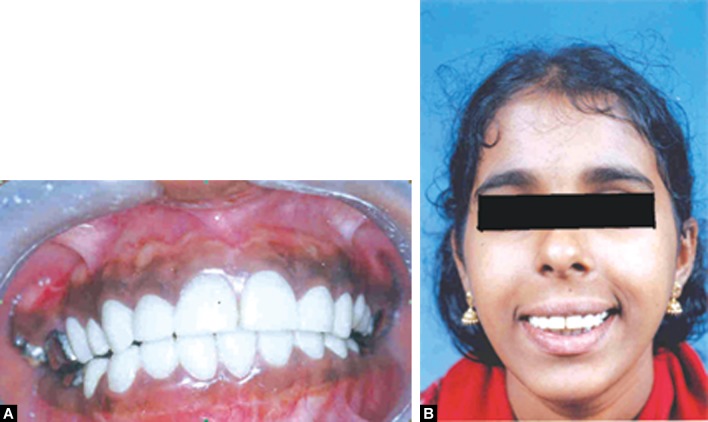
Post-treatment

Follow-up and clinical as well as radiographic evaluation was done at six-month intervals for one and half year and we observed that the post-treatment response was satisfactory (Fig. 10). The final phase of treatment with permanent occlusal rehabilitation will be done after the patient reaches adulthood.

## DISCUSSION

One of the most challenging dilemmas for a clinician is the treatment of a teenage patient with multiple defects of the enamel like amelogenesis imperfecta and its associated conditions. The affected enamel is disturbed in coloration, thickness and resistance. As a result these patients are more prone to tooth decay, periodontal disease and occlusal disturbances. In this manner amelogenesis imperfecta is a condition that seriously compromises the oral and psychological health of the patient and it requires early recognition and action.^8^ Nearly all patients affected with amelogenesis imperfecta assessed themselves to be aesthetically disturbed by their pretreatment condition. The condition negatively affected the relationship with other people and their self esteem. The prosthodontic management had a positive influence on all these patients and they all judged the aesthetic rehabilitation as the most important.^9^

The enamel in these children affected with the hypocalcified form of amelogenesis imperfecta is soft and cheesy and is easily removed exposing the dentinal tubules to bacterial invasion and pulpal or periapical pathosis like radicular cysts. There is also a loss in vertical dimension of occlusion as the enamel abrades of easily. Shear^10^ had reported that more than one radicular cyst may be found in a patient and this had led many authors to believe that there are cyst prone individuals who show a particular susceptibility to develop radicular cysts. It is possible that the immune mechanism may inhibit cyst formation in most individuals and cyst prone subjects have a defective surveillance and suppressive mechanism. 10,11 It is also possible that some individuals have a genetic tendency to develop radicular cysts.^10^ Multiple radicular cysts have also been associated with Basal Cell Nevus syndrome.^12^

Here we have rehabilitated a child having amelogenesis imperfecta with multiple periapical cysts who had undergone inadequate early intervention and management, which if left untreated would have resulted in osteomylitis, cellulitis 13 or even squamous cell carcinoma^13^,^14^further compromising the health of the child. The goal of treatment in this patient was to reduce the pathosis in the periapical region, improve the periodontal health, to protect and preserve the tooth structure and to establish an aesthetic appearance and efficient masticatory function until adulthood.

Hunter L and Stone D^15^ managed a nine-year-old boy by using superoccluding cobalt chrome onlays to increase the vertical dimension of occlusion .These restorations are used before the teeth are fully erupted. The use of the cast restorations both controls sensitivity and protects and preserves tooth structure. 15 Abdulkadir Sengun^1^ managed a 14-year-old boy with X linked recessive hypomaturation type of amelogenesis imperfecta with the placement of nickel chrome onlays in the molar region and direct resin composite restoration on the incisors, canines and premolars. However, the etching pattern of amelogenesis imperfecta is atypical due to abnormal prism structures^16^ so pretreatment of the affected enamel by sodium hypochlorite solution to enhance bonding has been suggested.^17^ Full coverage adhesive composite crowns or polycarbonate crowns have also been advocated but strict oral hygiene measures need to be implemented because of the plaque retentive nature of the restorative materials and passive eruption, which will inevitably expose more defective enamel.^6^

Scott H Rosenblum^7^ treated a 13-year-old with full coverage stainless steel crowns on the molars with an increase in vertical dimension and stainless steel crowns with veneer phasing on the anterior teeth. In adolescents, porcelain veneers are also likely to be useful; however their use with amelogenesis imperfecta has not been extensively reported.^18^ Porcelain jacket crowns which provide esthetic permanent restorations , have reportedly been successful in affected adults , but their use in young patients is contraindicated because of the presence of large pulp chamber and the likely need for frequent replacement due to passive eruption. 18

## CONCLUSION

The technical difficulties of treating these patients are far outweighed by the psychosocial benefits to the affected child. In conclusion, early intervention of treatment in these children affected with amelogenesis imperfecta is desirable before severe destruction can occur. As a pediatric dentist, we have to be aware that early intervention and preventive care is emphasized and that these children need constant restorative and occlusal rehabilitative alterations till the growth is completed.
